# Telitacicept demonstrates high probability of efficacy in myasthenia gravis: a Bayesian real-world study

**DOI:** 10.3389/fneur.2025.1736049

**Published:** 2026-01-27

**Authors:** Xinyi Duan, Haibing Xiao

**Affiliations:** 1 Shenzhen University Medical School, Shenzhen University, Shenzhen, Guangdong, China; 2 Department of Neurology, Neuromedicine Center, The University of Hong Kong-Shenzhen Hospital, Shenzhen, Guangdong Province, China; 3 Shenzhen Clinical Research Center for Rare Diseases, Shenzhen, China

**Keywords:** Bayesian analysis, efficacy and safety, myasthenia gravis, real-world study, telitacicept

## Abstract

**Objective:**

This real-world study evaluated the efficacy and safety of telitacicept, a dual BAFF/APRIL inhibitor, in patients with generalized myasthenia gravis (MG).

**Methods:**

In this retrospective study, 17 myasthenia gravis patients on stable background therapy received weekly subcutaneous telitacicept (160–240 mg). Efficacy was evaluated at 12/24 weeks using a primary composite endpoint (≥2-point MG-ADL and ≥3-point QMG reduction). A pre-specified Bayesian analysis, updating a conservative prior with observed data, was employed to determine the posterior probability of treatment success and its 95% credible interval. Safety and steroid-sparing effects were also assessed.

**Results:**

Of the 15 patients evaluable for efficacy (≥12 weeks treatment), 12 (80.0%) met the composite efficacy endpoint. Significant improvements were observed: mean MG-ADL decreased from 8.0 ± 4.4 to 4.2 ± 3.1 (*p* < 0.01); mean QMG decreased from 13.8 ± 5.5 to 7.6 ± 4.3 (*p* < 0.01). A robust steroid-sparing effect was demonstrated: the mean daily prednisone dose decreased by 76.0% (10.41 ± 7.30 mg to 2.50 ± 3.21 mg, *p* < 0.05), with 3 patients achieving complete withdrawal. Five patients attained Minimal Symptom Expression. Bayesian analysis yielded a posterior mean efficacy rate of 66.67% (95% CrI: 49.99–81.43%), with a probability (P) of exceeding 50% efficacy at 97.49%. Treatment was well-tolerated: only mild, transient AEs occurred (one injection-site reaction, one gastrointestinal event leading to withdrawal), and no serious adverse events (SAEs) were reported.

**Conclusion:**

This real-world study utilizing Bayesian analysis provides evidence supporting a high probability of efficacy for telitacicept in AChR-Ab-positive MG, demonstrating significant symptom improvement, substantial steroid-sparing effects, and favorable safety. These findings complement prior randomized controlled trial data and support the use of telitacicept in clinical practice. Study limitations include retrospective design and small sample size.

## 1 Introduction

Myasthenia Gravis (MG) is an autoimmune disorder mediated by autoantibodies targeting the acetylcholine receptor (AChR) at the neuromuscular junction, characterized by skeletal muscle weakness and fatigability (1, 2). The core pathological mechanism involves antibody-mediated blockade of neuromuscular signal transmission. The annual incidence of MG is estimated at 8–10 per million person-years, with a global prevalence of approximately 150–250 per million (3). While corticosteroids and azathioprine remain foundational immunosuppressants in myasthenia gravis (MG) management, their broad immunomodulatory mechanisms contribute to dose-limiting toxicities and cumulative organ damage in chronic use (4).

Building upon an enhanced understanding of MG pathogenesis, novel immunotherapeutic strategies are continuously emerging. Novel biologic agents now offer targeted alternatives: B-cell depletion therapies (anti-CD20 rituximab and anti-CD19 inebilizumab), FcRn antagonists (efgartigimod/rozanolixizumab), and complement inhibitors (eculizumab/ravulizumab) have demonstrated clinical efficacy with distinct safety profiles (5, 6). However, current B-cell-depleting agents still face challenges related to therapeutic fluctuations and long-term safety profiles (7, 8).

Telitacicept is a novel recombinant fusion protein that ameliorates symptoms by simultaneously blocking two key ligands regulating B-cell development—B lymphocyte stimulator (BLyS) and a proliferation-inducing ligand (APRIL). This dual inhibition suppresses aberrant B-cell differentiation and pathogenic autoantibody production, especially antibodies targeting AChR (9). This agent has demonstrated efficacy in systemic lupus erythematosus (SLE) and rheumatoid arthritis (RA), and received FDA orphan drug designation for MG treatment in 2022 (10–12).

Preliminary results from a randomized controlled trial (RCT) investigating telitacicept for MG indicated favorable efficacy and safety (13). However, given the substantial heterogeneity inherent in MG, the strictly controlled RCT data require further validation of its effectiveness and safety profile in environments more closely mirroring clinical practice. Real-world studies (RWS) are uniquely positioned to provide comprehensive evidence complementary to RCTs (14).

Therefore, this study aims to analyze the clinical utility of telitacicept in a series of 17 MG patients based on real-world data (RWD), assessing its impact on symptom alleviation, quality of life (QoL) improvement, and safety. This research will provide clinical evidence for the role of telitacicept within the MG therapeutic armamentarium and serve as an important complement to prior RCT findings.

## 2 Method

### 2.1 Study design

This study was a real-world data (RWD)-based case series designed to evaluate the efficacy and safety of Telitacicept in patients with myasthenia gravis (MG). We retrospectively collected clinical data from patients before and after Telitacicept treatment to assess its clinical efficacy and safety profile.

### 2.2 Study population

Seventeen MG patients admitted to our hospital between June 2023 and June 2024 were enrolled, provided they met the predefined inclusion and exclusion criteria.

**Inclusion Criteria:**

1. **Age:** ≥ 18 years.
2. **Diagnosis:** Met the diagnostic criteria for MG as defined in the “Management of Myasthenia Gravis Around the Globe: Consensus Guidelines Versus Realities of Practice” (15), confirmed by repetitive nerve stimulation (RNS) testing and/or positive AChR antibody (AChR-Ab) serology.
3. **Treatment Regimen:** Received standard subcutaneous administration of Telitacicept (either 160 mg or 240 mg once weekly).
4. **Informed Consent:** Provided written informed consent after full comprehension of the study protocol.

**Exclusion Criteria:**

1. **Active Infection:** Active infection requiring systemic antimicrobial therapy within the 4 weeks prior to enrollment (including, but not limited to, active tuberculosis, hepatitis B, hepatitis C, HIV infection, and herpes zoster).
2. **Significant Organ Dysfunction:**

- Liver: Alanine aminotransferase (ALT) or aspartate aminotransferase (AST) levels > 2.5 times the upper limit of normal (ULN).
- Kidney: Serum creatinine (Cr) level > 1.5 times ULN.

- **Other Severe Comorbidities:** Uncontrolled malignancy, thymoma, severe cardiovascular or cerebrovascular disease, hematopoietic system disorders, or other significant systemic diseases deemed by the investigator as potentially interfering with study outcomes.
- **Other Factors:** History of substance abuse, severe psychiatric disorders hindering compliance, pregnancy or lactation, or other conditions judged by the investigator to prevent study completion or pose additional risks.

And it is necessary to emphasize that Serum AChR/MUSK antibodies in the patients were detected using the classic Radioimmunoassay (RIA). Kits were procured from RSR Ltd. (United Kingdom, Cat. No.: RBA/100) and assays were performed strictly according to the manufacturer’s instructions. The positive threshold was defined as >0.5 nmol/L. All analyses were conducted at Hangzhou CRED DIAGNOSTICS Intelligence Medical Laboratory Co., Ltd., which holds ISO 9001:2015 quality certification.

### 2.3 Outcome measures and follow-up

All patients received subcutaneous Telitacicept injections once weekly, initiated at 160 mg. Thirteen patients remained on this dose, while four had their dose escalated to 240 mg due to insufficient clinical response. Patients underwent weekly clinic visits throughout the treatment period for efficacy and safety monitoring. Primary efficacy assessment timepoints were set at 12 and 24 weeks. The assessment of ADL and QMG was conducted at least 8 h after the last administration of pyridostigmine bromide to rule out the influence of transient cholinergic effects (15). The primary composite efficacy endpoint was defined as simultaneously achieving a reduction of ≥ 2 points in the Myasthenia Gravis Activities of Daily Living (MG-ADL) score from baseline (representing the minimal clinically important difference threshold) *and* a reduction of ≥ 3 points in the Quantitative Myasthenia Gravis (QMG) score (9, 16). Consequently, data collected during each visit included MG-ADL score, QMG score, and changes in corticosteroid dose. *Note:* The MG-ADL scale assesses patients’ ability to perform daily activities, the QMG score quantifies muscle weakness severity, and the Myasthenia Gravis Foundation of America (MGFA) classification categorizes overall disease status. Critically, “Baseline medication on regimen” refers to background immunosuppressive therapies required to be stable for at least 3 months prior to telitacicept initiation (with no changes in type, dosage, or frequency), whereas “Adjusted medication on regimen” denotes the modified dosages recorded at the 24-week endpoint (Table 1).

**Table 1:** Baseline characteristics of MG patients treated with TLR.

Serial number	Gender	Age (years)	Disease duration (years)	MG subtype	Antibody subtype	Thymectomy	Comorbidities	Baseline medication regimen	Adjusted medication regimen	Treatment course of TLR (weeks)
1	Male	36	3	3a	Anti-achr (+)	Y	Syphilis	PYR 60 mg TID, PRED 10 mg QD	PYR 60 mg BID	24
2	Female	35	1	2a	Anti-achr (+)	Y	/	PYR 60 mg TID	PYR 60 mg QD	24
3	Female	39	13	2a	Anti-achr (+)	N	/	TAC 3 mg QD, PYR 60 mg TID	PYR 60 mg TID	24
4	Female	45	32	3a	Anti-achr (+)	Y	Steroid-induced diabetes, Iron deficiency poverty	PYR 120 mg QID	PYR 120 mg QID	24
5	Female	37	19	2a	Anti-achr (+)	Y	Postoperative status of papillary thyroid carcinoma	MMF 0.5 g BID, PYR 60 mg TID	PYR 60 mg TID	24
6	Male	37	3	2a	Anti-Musk (+)	N	/	TAC 4 mg QD	/	24
7	Female	69	4	3a	Anti-achr (+)	N	Sleep disorders, Osteoporosis	PYR 60 mg TID	PYR 60 mg TID	12
8	Male	61	1	2a	Anti-achr (+)	N	Gout, Hypertension	TAC 3 mg QD, PYR 60 mg QID, PRED 10 mg QD	TAC 2 mg QD, PYR 60 mg BID, PRED 5 mg QD	24
9	Male	47	4	2a	Anti-achr (+)	N	Hypertension, psoriasis	PYR 120 mg TID, PRED 20 mg QD	PYR 120 mg TID	24
10	Male	26	10	2a	Anti-achr (+)	Y	/	PYR 60 mg TID, TAC 1 mg QD, PRED 5 mg QD	PYR 60 mg TID, TAC 2 mg QD, PRED 5 mg QD	24
11	Male	42	5	2a	Anti-achr (+)	Y	/	MMF 0.75 g BID, PYR 60 mg QID	MMF 0.5 g BID, PYR 60 mg BID	12
12	Female	57	20	2a	Anti-achr (+)	N	/	PYR 30 mg TID, PRED 7.5 mg QD	PYR 30 mg BID	24
13	Female	38	8	3a	Anti-achr (+)	N	Iron deficiency and poverty, Hip osteoarthritis, Systemic lupus erythematosus, Osteoporosis	PYR 120 mg TID, TAC 2 mg BID, HCQ 0.2 g QD, PRED 10 mg QD	PYR 120 mg BID, TAC 1.5 mg BID, HCQ 0.4 g QD, PRED 5 mg QD	12
14	Female	35	4	3a	Anti-achr (+)	Y	Iron deficiency and poverty	PYR 60 mg QID, TAC 1.5 mg QD, PRED 5 mg QD	PYR 60 mg QID + TAC 1.5 mg QD, PRED 5 mg QD	12
15	Female	41	10	2a	Total negative antibody	Y	/	PYR 60 mg TID	PYR 60 mg TID	1
16	Male	32	27	2a	Anti-achr (+)	N	/	PYR 60 mg QID	PYR 60 mg BID	24
17	Female	28	20	2a	Anti-achr (+)	Y	/	PYR 120mgTID	PYR 120 mg QD	2

PYR, Pyridostigmine; TAC, Tacrolimus; MMF, Mycophenolate mofetil; TLR, Telitacicept; PRED, prednisone; HCQ, hydroxychloroquine; BID, twice daily; TID, three times daily; QID, four times daily; QD, once daily. Anti-achr (+): Acetylcholine receptor antibody positive; Anti-Musk (+): Muscle-specific kinase antibody positive; Total negative antibody: Seronegative MG.

### 2.4 Safety assessment

Safety was assessed by recording the incidence and severity of adverse events (AEs). At each visit, investigators documented all patient-reported adverse reactions, including injection site reactions and gastrointestinal disturbances. Serious adverse events (SAEs) were defined as events resulting in hospitalization, disability, or life-threatening conditions. All AEs were meticulously recorded. Analyses included AE incidence rate (graded according to CTCAE v5.0), SAE incidence rate, and the nature of reported events.

### 2.5 Statistical analysis

Continuous data underwent normality testing using the Shapiro–Wilk test. Normally distributed variables are presented as mean ± standard deviation (SD). Within-group comparisons over time (e.g., pre- vs. post-treatment) were performed using paired Student’s t-tests. Comparisons across multiple time points utilized repeated-measures analysis of variance (RM-ANOVA). Non-normally distributed data are expressed as median (interquartile range, IQR). Within-group comparisons over time used the Wilcoxon signed-rank test, while comparisons across multiple time points employed the Friedman test. Between-group comparisons for independent samples utilized the Mann–Whitney U test. Categorical variables are presented as frequency (percentage) and compared using Chi-square or Fisher’s exact tests as appropriate. AE incidence rates were summarized using descriptive statistics for safety evaluation. All statistical tests were two-sided, with a significance level set at *p* < 0.05. Analyses were conducted using SPSS software.

Additionally, Bayesian analysis was employed to evaluate the pre-specified composite efficacy endpoint defined as achieving both a ≥ 3-point reduction in QMG score and a ≥ 2-point reduction in MG-ADL score concurrently (17). A Beta prior distribution was constructed based on historical placebo-response data derived from a study involving 16 patients (18), specifically chosen to represent skepticism of benefit, with shape parameters set to *α* = 10 and *β* = 8 (equivalent to 10 successes out of 18 hypothetical trials). This prior was then updated by combining it with the observed treatment success rate from the current study cohort (*n* = 15, with 12 successes), resulting in posterior distribution parameters of *α* = 22 (10 + 12) and *β* = 13 (8 + (15–12)). We report the posterior mean success probability, its 95% credible interval (CrI), and relevant posterior probabilities (e.g., P (posterior success rate > target threshold)). Bayesian analyses were performed using R statistical software (version 4.5.1). Data visualization and posterior distribution modeling utilized packages including ggplot2 and bayesplot.

### 2.6 Ethical statement

This study received approval from the Institutional Review Board of Ethics Committee of The University of Hong Kong-Shenzhen Hospital prior to initiation. Written informed consent was obtained from all participating patients. The study was conducted in strict accordance with the ethical principles outlined in the Declaration of Helsinki and the International Council for Harmonization’s Guideline for Good Clinical Practice (ICH-GCP), ensuring the protection of patient rights throughout the research process.

## 3 Results

### 3.1 Baseline clinical characteristics

Among the 17 gMG patients treated at our institution, 88% (n = 15) received at least 12 weeks of Telitacicept injections, and 11 patients completed the full 24 weeks of treatment. Baseline clinical characteristics are summarized in Table 1. The cohort comprised 7 males (41.2%) and 10 females (58.8%), with a mean age of 41.47 ± 11.47 years (range: 26–69 years) and a median disease duration of 8 years (IQR: 3.5–19.5). Fifteen patients (88.2%) were AChR-Ab positive, one patient (5.9%) was MuSK-Ab positive, and one patient (5.9%) was seronegative (antibody-negative). Nine patients (52.9%) had undergone thymectomy, with pathological findings showing thymic hyperplasia in 8 cases and unknown histology in one case. According to the MGFA clinical classification, 12 patients (70.6%) were Class IIa, and 5 patients (29.4%) were Class IIIa. (Table 1).

Comorbidities included hypertension (*n* = 2, 11.8%), steroid-induced diabetes (*n* = 1, 5.9%), iron-deficiency anemia (*n* = 3, 17.6%), osteoporosis (*n* = 3, 17.6%), history of papillary thyroid carcinoma (*n* = 1, 5.9%), gout (*n* = 1, 5.9%), systemic lupus erythematosus (SLE; *n* = 1, 5.9%), and syphilis (*n* = 1, 5.9%; Table 1).

At baseline, patients exhibited diverse concomitant immunosuppressive regimens. Twelve patients (80.0% of the 15 analyzed) received Telitacicept combined with at least one other immunosuppressant. Glucocorticoids (GCs) were the most common concomitant therapy (*n* = 7, 46.7%), followed by tacrolimus (*n* = 6, 40.0%) and mycophenolate mofetil (MMF; *n* = 2, 13.3%). Three patients were receiving both tacrolimus and GCs concurrently. (Table 1).

### 3.2 Clinical efficacy of telitacicept

During the study period, the majority of evaluated patients achieved significant clinical improvement. Specifically, 12 out of 15 patients (80.0%) met the pre-specified dual composite endpoint criteria: a reduction of ≥2 points in the MG Activities of Daily Living (MG-ADL) score (representing the Minimal Clinically Important Difference, MCID) concomitant with a reduction of ≥3 points in the Quantitative Myasthenia Gravis (QMG) score.

**Figure 1:**
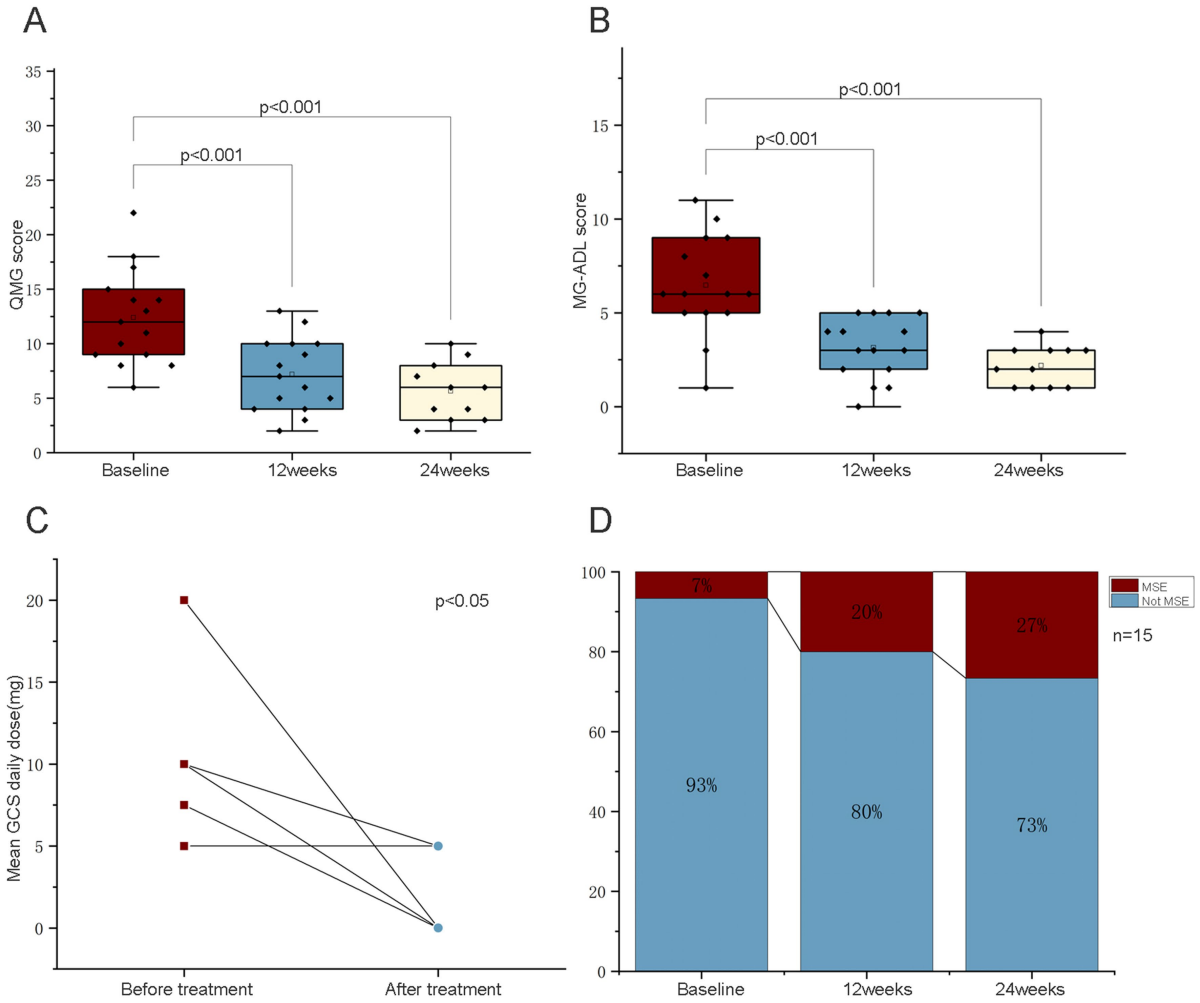
**(A)** Mean change from baseline to week 24 in MG-ADL score (mean ± SD). **(B)** Mean change from baseline to week 24 in QMG score (mean ± SD). **(C)** Reduction in mean daily prednisone dose. **(D)** Proportion of patients in achieving minimal symptom expression (MSE).

Patient symptom burden significantly decreased as evidenced by reductions in both key scales: the mean MG-ADL score decreased from 8.0 ± 4.4 at baseline to 4.2 ± 3.1 (*p* < 0.01), and the QMG score decreased from 13.8 ± 5.5 to 7.6 ± 4.3 (*p* < 0.01; Figures 1A,B; Table 2). A significant steroid-sparing effect was observed. Among the 7 patients using prednisone at baseline (46.7%).

**Table 2:** Longitudinal changes in MG-ADL and QMG scores during TLR treatment.

Serial number	MG-ADL score			QMG score		
	Baseline	Week 12	Week 24	Baseline	Week 12	Week 24
1	10	5	3	18	12	9
2	7	5	2	13	10	6
3	6	1	1	14	6	4
4	11	5	4	22	13	10
5	6	4	3	11	9	7
6	9	4	3	17	10	8
7	8	3		15	8	
8	6	3	1	9	4	3
9	5	2	1	8	3	2
10	6	4	3	8	5	3
11	1	0		6	2	
12	5	2	2	12	7	6
13	9	3		14	5	
14	8	7		13	13	
15^*^	6	6		12	12	
16	3	1	1	9	4	4
17*	5	5		10	10	

MG-ADL, Myasthenia Gravis Activities of Daily Living; QMG, Quantitative Myasthenia Gravis. *Patients who discontinued the study early and did not complete the 12-week and/or 24-week efficacy assessments.

The mean daily prednisone dose was reduced from 10.41 ± 7.30 mg to 2.50 ± 3.21 mg (*p* < 0.05), representing an absolute mean reduction of 7.91 mg and a relative reduction of 76.0% (Figure 1C).

Three patients (42.9%) achieved complete GC withdrawal (prednisone dose = 0 mg). Five patients (33.3%) attained Minimal Symptom Expression (MSE), with three achieving this status within 12 weeks and two by 24 weeks (Figure 1D). Notably, among patients who did not reach MSE (Patients 1, 12), the mean daily prednisone dose still decreased. Furthermore, Patients 2, 3, 4, 5, 6, 7, 11 and 16 remained off GC therapy throughout the study period. Friedman test analysis confirmed a statistically significant reduction in mean daily prednisone dose over the study (*p* < 0.05), establishing evidence for a significant steroid-sparing effect of telitacicept.

**Figure 2:**
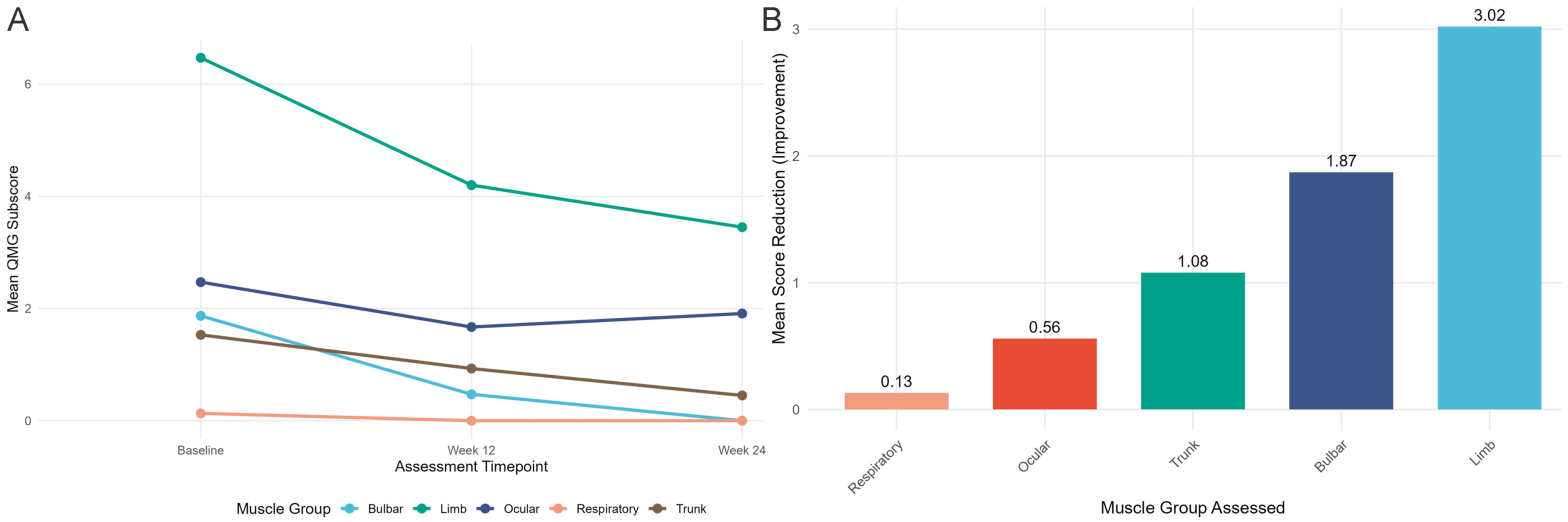
Change in muscle-specific weakness with telitacicept treatment in patients with myasthenia gravis. **(A)** Line graph showing the mean Quantitative Myasthenia Gravis (QMG) subscore for each specific muscle group (ocular, limb, trunk, bulbar, and respiratory) at baseline, week 12, and week 24 of treatment. Lower scores indicate improvement in muscle strength. A consistent downward trend is observed across all muscle groups, with the bulbar and respiratory scores reaching zero by week 24. **(B)** Bar graph illustrating the mean improvement in QMG subscore (calculated as baseline score minus week 24 score) for each muscle group. The greatest improvement was observed in limb muscles (mean reduction: 3.02 points), followed by bulbar muscles (1.87 points), trunk muscles (1.08 points), ocular muscles (0.56 points), and respiratory muscles (0.13 points).

And the longitudinal analysis of muscle-specific weakness revealed a consistent pattern of improvement across all assessed domains (Figure 2). The mean QMG subscores demonstrated progressive reduction from baseline to weeks 12 and 24 for ocular (2.47 to 1.67 to 1.91), limb (6.47 to 4.20 to 3.45), trunk (1.53 to 0.93 to 0.45), bulbar (1.87 to 0.47 to 0.00), and respiratory muscles (0.13 to 0.00 to 0.00), with bulbar and respiratory functions achieving complete resolution in the cohort by week 24 (Figure 2A). The magnitude of improvement varied across muscle groups (Figure 2B). Limb muscles showed the greatest mean improvement (3.02 points), followed by bulbar (1.87 points), trunk (1.08 points), ocular (0.56 points), and respiratory muscles (0.13 points).

**Figure 3:**
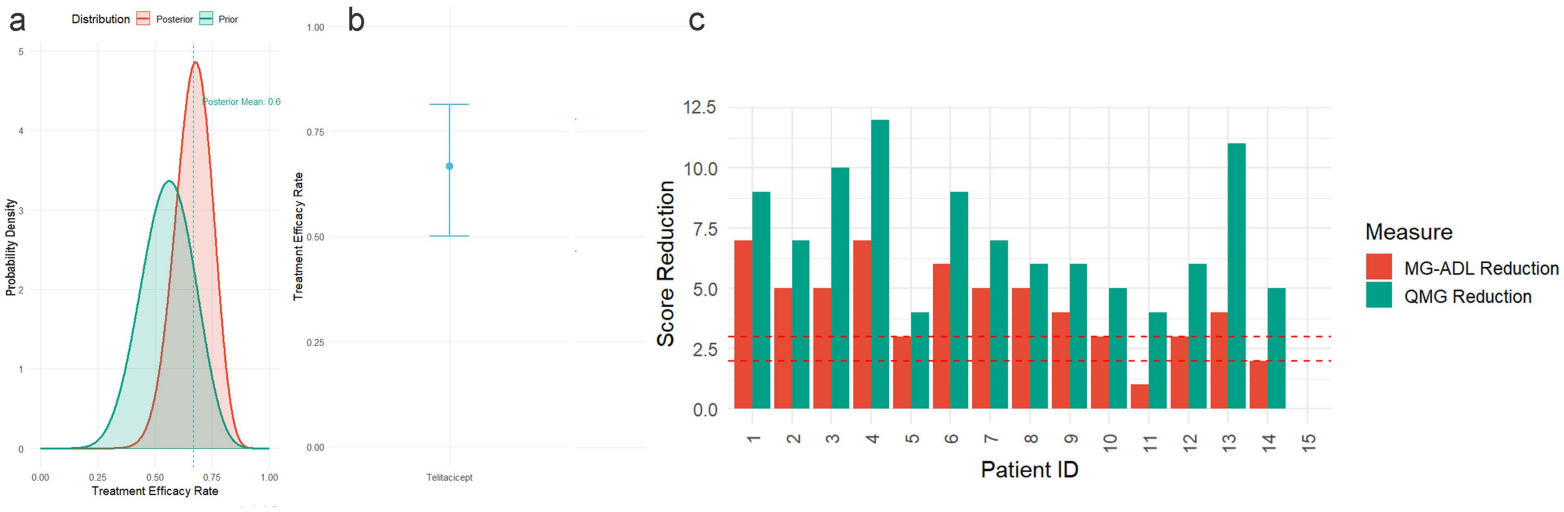
**(a)** Prior and posterior distribution comparison: Blue curve represents the prior distribution based on historical data (Beta(10, 8)); Red curve represents the posterior distribution combining historical and current data (Beta(22, 11)); Vertical dashed line indicates the posterior mean (0.629); Shaded area represents the 95% credible interval. **(b)** Posterior efficacy rate estimate for Telitacicept: Point estimate represents the posterior mean (0.6667), error bars represent the 95% credible interval (0.4999–0.8143). **(c)** Individual patient QMG and MG-ADL score changes: Bar chart displays QMG and MG-ADL score changes for each patient; Horizontal dashed lines indicate clinical significance thresholds (QMG ≥ 3, MG-ADL ≥ 2).

**Figure 4:**
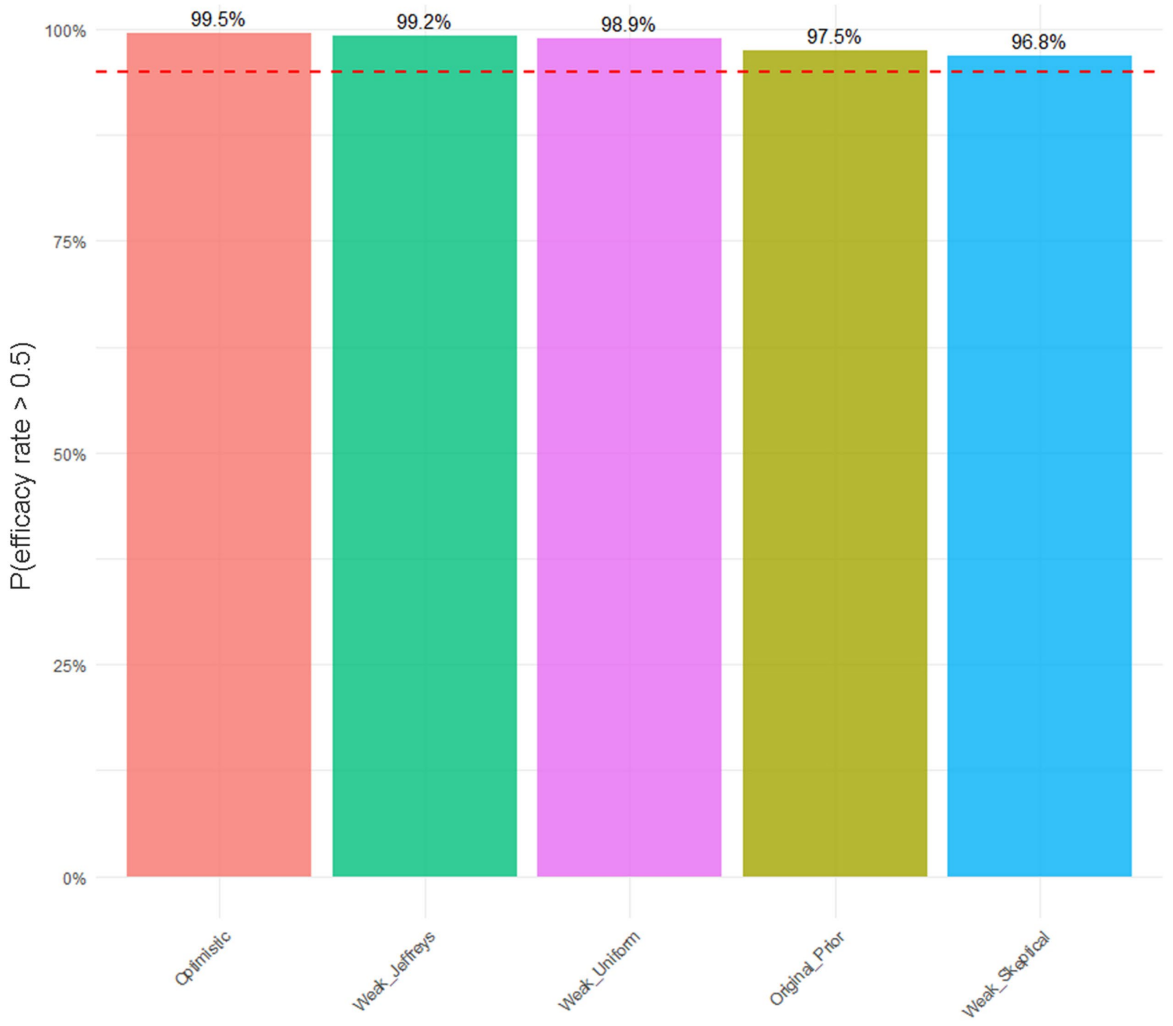
Sensitivity analysis: Robustness of P(efficiency > 50%) under different prior distributions. The red dashed line represents the 95% probability threshold.

To further contextualize these efficacy results, a Bayesian analysis was employed, incorporating historical data from a prior study involving 16 patients where the composite endpoint was achieved in 9 patients (56.3%; Figure 3c). Using a Beta(10,8) prior distribution (reflecting prior skepticism based on this historical cohort), and updating it with the observed response data from this study (12 responders among 15 evaluable patients; specific responder count critical for posterior calculation), the posterior distribution yielded a mean success rate of 0.6667 (Figure 3a) with a 95% credible interval (CrI) of 0.4999–0.8143 (Figure 3b). This indicates a 95% probability that the true response rate to Telitacicept lies between 49.99 and 81.43%. Moreover, the posterior probability of the response rate exceeding 50% was 97.49% (P (rate > 0.5 | Data) = 0.9749) and exceeding 60% was 79/54% (P(rate > 0.6 | Data) = 0.7954; Table 3). Meanwhile, to assess the sensitivity of our Bayesian analysis results to prior distribution selection, we conducted a suite of robustness analyses employing multiple priors, including weakly informative priors and priors with different parameter specifications. The findings demonstrate that the efficacy estimates of telitacicept remained consistent across all tested prior configurations. (Figure 4).

**Table 3:** Bayesian analysis summary of telitacicept treatment efficacy.

Metric	Value
Historical data–effective patients	9/16 (56.3%)
Current study–effective patients	12/15 (80.0%)
Prior distribution parameters (α, β)	(10, 8)
Posterior distribution parameters (α, β)	(22, 11)
Posterior mean	0.6667
95% credible interval	(0.4999, 0.8143)
P(efficacy rate > 0.5)	0.9749
P(efficacy rate > 0.6)	0.7954
P(efficacy rate > 0.7)	0.3560

### 3.3 Safety

Safety was assessed in the full enrolled cohort of 17 patients. Fifteen patients, who completed at least 12 weeks of therapy, were included in the efficacy analysis set. Two patients were excluded from the efficacy analysis: Patient No. 15 experienced stomach discomfort after taking one dose of telitacicept and then withdrew from the study. Patient No. 17 withdrew after the second administration due to having a personal plan for pregnancy (no treatment-related adverse events occurred).

No serious adverse events (SAEs) occurred during the study. Treatment-emergent adverse events (TEAEs) included mild, transient injection-site reactions observed in one patient and mild, transient gastrointestinal distress reported by the patient who subsequently withdrew due to this event. All adverse events resolved spontaneously within a few days. No severe adverse events were reported.

## 4 Discussion

In this real-world investigation of patients with myasthenia gravis (MG), telitacicept demonstrated significant clinical efficacy and a favorable safety profile. A key methodological strength underpinning these conclusions is the application of a Bayesian analytical framework. This approach was deliberately chosen to enhance statistical power and mitigate the inherent limitations of a small sample size, a common challenge in real-world evidence generation (19). By formally integrating robust prior evidence from registrational trials with our contemporary data, the Bayesian model yields more precise and credible efficacy estimates (20, 21). To formally integrate prior knowledge, we established a conservative Beta (10,8) prior distribution based on historical data where the response rate was 56.25%. Upon updating with our compelling observed data—wherein 12 of 15 patients (80.00%) met the primary composite endpoint—the model yielded a posterior mean response rate of 66.67% with a 95% credible interval of [49.99, 81.43%]. Crucially, this translates into a 97.49% posterior probability that the true response rate exceeds the clinically significant 50% threshold, and a 79.54% probability of it exceeding 60%. This probabilistic conclusion offers a more nuanced and clinically informative alternative to conventional frequentist analysis. Furthermore, sensitivity analyses confirmed that these efficacy estimates remained robust across various prior specifications, bolstering the reliability of our findings (22). Notably, since 37% of patients in the historical placebo cohort had thymoma (vs. 0% in our cohort), we rigorously addressed this heterogeneity through Bayesian sensitivity analyses. When replacing the historical-data prior with a non-informative prior (Beta (1,1)) and a skeptical prior (centered at *θ* < 0.3 with equivalent sample size), the posterior probability of an efficacy rate exceeding 50% remained consistently high (>90% in all scenarios, Figure 3). This demonstrates that the conclusions are robust across different prior assumptions. Per FDA Bayesian guidance (23, 24), such insensitivity to prior specification confirms that our findings are predominantly driven by the observed study data rather than external sources. Clinical interpretations should therefore prioritize the current trial’s evidence. Therefore, the substantial symptomatic improvements observed in our cohort are supported by a rigorous and intentionally chosen analytical strategy.

Efficacy assessments revealed significant improvements in myasthenic symptoms. The substantial decline in MG-ADL scores (mean reduction: 3.8 points from baseline 8.0 ± 4.4 to 4.2 ± 3.1) demonstrated telitacicept’s capacity to alleviate functional impairment in daily activities. This reduction exceeded the minimum clinically important difference (MCID) threshold (≥2 points), effectively transitioning patients from “mild functional limitation” (MG-ADL > 5) to “near-normal daily living” (MG-ADL < 5). The objective clinical response rate (80.0% achieving ≥5-point QMG reduction—meeting guideline-defined moderate improvement) closely approached outcomes from registrational Phase II trials (92.9% at 160 mg; 100% at 240 mg achieving ≥3-point QMG reduction), underscoring telitacicept’s applicability despite the heightened complexity of our cohort (45% post-thymectomy; 70.6% requiring multi-drug immunosuppressive regimens). And our core efficacy data demonstrate that telitacicept broadly improves function across multiple muscle groups, with the most pronounced improvement observed in limb and bulbar muscles. This finding is consistent with the comprehensive enhancement in patients’ daily living activities (as measured by the MG-ADL score) and the significant steroid-sparing effect.

Critically, glucocorticoid burden markedly decreased during telitacicept therapy. The mean daily prednisone dosage declined by 76.0% (10.41 ± 7.30 mg to 2.50 ± 3.21 mg), with three patients achieving sustained zero-dose maintenance. This steroid-sparing effect carries profound implications: It mitigates exposure to endocrine-related comorbidities such as osteoporosis (prednisone >7.5 mg/day increases fracture risk by 2-fold) while enhancing treatment adherence and quality of life. Telitacicept’s metabolic safety advantages over conventional immunosuppressants (e.g., tacrolimus-associated hyperglycemia and nephrotoxicity) further position it as an optimal complement to glucocorticoid minimization strategies (25, 26). Reduced dependence on traditional agents may benefit patients with treatment-refractory profiles (27).

Notably, the profound clinical amelioration observed in our MuSK antibody-positive patient (MG-ADL: 9 → 3; QMG: 17 → 8) provides critical mechanistic insights into managing this distinct serotype. Unlike AChR-MG, MuSK-MG is characterized by IgG4 autoantibodies which exert pathogenicity by blocking MuSK-LRP4 interaction independent of complement activation, thereby rendering C5 inhibitors ineffective. Furthermore, recent evidence suggests MuSK-MG is predominantly driven by high-turnover short-lived plasmablasts rather than long-lived plasma cells (9, 28). The observed efficacy likely stems from telitacicept’s dual BLyS/APRIL blockade: by suppressing nascent plasma cell generation and depriving cells of APRIL—a critical survival factor—it accelerates the apoptosis of these short-lived IgG4-secreting plasmablasts inherent to MuSK-MG. This upstream suppression directly halts pathogenic antibody production, bypassing the irrelevant complement pathway and providing a precise therapeutic rationale for this often-refractory subtype (29–31).

Interindividual efficacy variation observed in our cohort, potentially modulated by disease chronicity and immunophenotypic factors, necessitates personalized therapeutic strategies. Dose escalation to 240 mg achieved minimal symptom expression in 2/4 patients, further suggesting the value of protocol individualization (18).

Safety outcomes aligned with telitacicept’s established autoimmune disease profile. All adverse events were mild-to-moderate and manageable without intervention (32). Telitacicept demonstrated a stable and favorable safety profile in this short-term real-world cohort (33, 34) with no severe infections, hypogammaglobulinemia, or autoimmune exacerbations observed. One discontinuation (transient gastric intolerance, possibly mediated via APRIL intestinal signaling) and one self-limiting injection-site reaction were the only tolerability concerns. Vigilance remains warranted regarding latent infection reactivation in high-risk patients (one case involved historical syphilis with preserved serostatus), emphasizing the need for pretreament screening and monitoring.

Emerging molecular therapies delineate distinct management strategies focusing on either rapid rescue or sustained disease modification. FcRn and complement inhibitors are characterized by a rapid onset of action suitable for managing myasthenic crisis by targeting downstream effectors or circulating IgG (35). Conversely, telitacicept targets the upstream survival of plasma cells to address the fundamental source of autoimmunity. This mechanism supports long-term disease stabilization and deep corticosteroid tapering as evidenced by the 76% dose reduction in our cohort. Furthermore, telitacicept offers durable remission without the pulsatile fluctuations or antibody rebound risks associated with cyclic FcRn inhibitor regimens (36). Consequently, telitacicept occupies a critical therapeutic niche as a broad-spectrum and steroid-sparing maintenance agent.

Although our findings are encouraging, several limitations warrant cautious interpretation. Firstly, the retrospective nature of the study carries the potential for selection bias; consequently, prospective studies are required to validate the generalizability of our conclusions (37). Secondly, the current follow-up duration was insufficient to evaluate long-term patterns of relapse, evolution of drug resistance, and sustainability of corticosteroid taper during extended therapy. Thirdly, the limited sample size (n = 15) and inadequate representation of crucial patient subgroups—such as those with ocular myasthenia gravis (MG), elderly patients (>65 years), and a sufficient number of MuSK antibody-positive (MuSK-Ab+) cases (only one was included)—restrict exploration of population heterogeneity. It should also be noted that the current study was limited by its small sample size and relatively short follow-up period (maximum 24 weeks), particularly for assessing rare but serious adverse events (SAEs) such as severe infections and hypogammaglobulinemia. Finally, the lack of systematic monitoring of target biomarkers (e.g., antibody titers, B-cell subset dynamics) limits inferences regarding the drug’s precise mechanisms of action. Future multi-center studies with expanded cohorts are urgently needed to strengthen the evidence base.

Collectively, by employing rigorous Bayesian methodology, this real-world study validates the clinical efficacy and safety of telitacicept in patients with MG, providing a more robust foundation for its broader clinical application. Future research should prioritize exploring personalized treatment strategies and accumulating long-term follow-up data. Efforts should focus on refining subtyping to identify optimal treatment candidates (e.g., thymoma-associated MG) and optimizing combination approaches (e.g., sequential use with FcRn inhibitors) to further enhance MG management.

## 5 Conclusion

This Bayesian real-world analysis confirms that telitacicept exhibits a high probability of clinical efficacy in AChR antibody-positive myasthenia gravis (MG) patients (posterior probability of response >50%: 0.9749%) alongside a favorable safety profile. The agent significantly improves daily living function and MG-specific symptom burden in the majority of treated patients, achieves clinically meaningful steroid-sparing effects, and demonstrates mild and manageable adverse events.

## Data Availability

The original contributions presented in the study are included in the article/supplementary material, further inquiries can be directed to the corresponding author.
